# Increasing utilisation of skilled facility-based maternal healthcare services in rural Zambia: the role of safe motherhood action groups

**DOI:** 10.1186/s12978-017-0342-1

**Published:** 2017-07-10

**Authors:** Cephas Sialubanje, Karlijn Massar, Larah Horstkotte, Davidson H. Hamer, Robert A.C. Ruiter

**Affiliations:** 1 Ministry of Health, Monze District Medical Office, P.O. Box 660144, Monze, Zambia; 2 Department of Work and Social Psychology, Maastricht University, P.O. Box 616, 6200MD Maastricht, The Netherlands; 3 Zambia Centre for Applied Health Research and Development, P.O. Box 30910, Lusaka, Zambia; 4 Centre for Global Health and Development Boston University School of Public Health, Crosstown 3rd floor, 801 Massachusetts Avenue, Boston, MA 02118 USA; 5 Department of Global Health, Boston University School of Public Health, Crosstown 3rd floor, 801 Massachusetts Avenue, Boston, MA 02118 USA

**Keywords:** Maternal health, Community interventions, Safe Motherhood Action Groups, Kalomo, Zambia

## Abstract

**Background:**

Community-centred health interventions, such as Safe Motherhood Action groups (SMAGs), have potential to lead to desired health behavioural change and favourable health outcomes. SMAGs are community-based volunteer groups that aim to reduce critical delays that occur at household level with regard to decision-making about seeking life-saving maternal care at health facilities. The aim of this study was to explore perspectives, roles, achievements and challenges of the SMAG programme in Kalomo, Zambia.

**Methods:**

In-depth interviews (IDIs) were conducted in 7 health centres in Kalomo district between 1st April and 20th May, 2015 with 46 respondents comprising 22 SMAG members, 5 headmen, 10 mothers, 3 husbands, 5 nurses, and 1 district maternal and child health coordinator. Perspectives on the selection, training, roles, achievements and challenges of the SMAG programme were explored.

**Results:**

Respondents were aware of the presence, selection, training and roles of the SMAG members and had a positive attitude towards the programme. They believed that the SMAG programme led to an increase in women’s risk perception about pregnancy and childbirth-related complications. Further, participants believed that the programme resulted in increased utilisation of facility-based antenatal, delivery and postnatal care, and improvement in maternal and newborn health outcomes. However, various challenges affected implementation of the SMAG programme. Among these were insufficient material and financial support to the programme, lack of refresher training for SMAG members, poor quality of care in health care facilities due to a lack of maternity waiting homes, low staffing levels in health facilities, the poor state and small size of the labour wards, and lack of equipment to handle obstetric emergencies.

**Conclusion:**

The SMAG programme has potential to be an important community intervention for increasing utilisation of facility-based skilled care and improving maternal and newborn health outcomes.

## Plain English summary

Safe Motherhood Action Groups (SMAGs) are community-based volunteer groups that aim to reduce critical delays that occur at household level with regard to decision-making about seeking life-saving maternal healthcare at health facilities. The aim of this study was to explore perspectives, roles, achievements and challenges of the SMAG programme in Kalomo, Zambia. We conducted 46 in- depth interviews in 7 health centres between 1st April and 20th May, 2015. Participants comprised 22 SMAG members, 5 headmen, 10 mothers, 3 husbands, 5 nurses and 1 district maternal and child health coordinator. Respondents were aware of the presence, selection, training and roles of the SMAG members and had a positive attitude towards the programme. They believed that the SMAG programme led to an increase in women’s risk perception about pregnancy and childbirth-related complications. Further, participants believed that the programme resulted in increased utilisation of facility-based antenatal, delivery and postnatal care, and improvement in maternal and newborn health outcomes. However, various challenges affected implementation of the SMAG programme. Among these were insufficient material and financial support to the programme, lack of refresher training for SMAG members, poor quality of care in health care facilities due to a lack of maternity waiting homes, low staffing levels in health facilities, the poor state and small size of the labour wards, and lack of equipment to handle obstetric emergencies. Thus, the SMAG programme might be an important intervention for increasing utilisation of facility-based skilled care and improving maternal and newborn health outcomes.

## Background

Limited access to and low utilisation of skilled birth attendance are some of the major reasons for the high maternal mortality ratio (MMR) in Zambia, currently at 398 deaths per 100,000 live births [1–6]. Several factors including women’s lack of decision-making capacity, low socioeconomic status and dependency on their husbands for financial support, long distances to health care facilities, high transportation costs, logistical challenges and low quality of care in rural Zambia limit access to facility-based delivery services and cause many women (53%) to give birth at home [7–13].

To increase the utilisation of maternal healthcare services (MHS), in 2003 the Zambian Ministry of Health (MOH) established Safe Motherhood Action Groups (SMAGs) as part of a national safe motherhood programme [14]. The SMAGs are community-based volunteer groups that aim to reduce critical delays − including the delay for women to seek MHS, delay to reach the healthcare facility, and delay to access or receive quality MHS from a skilled provider [15]−that prevent women from seeking and accessing life-saving MHS provided at health facilities [14–16]. SMAGs comprise various community volunteers such as traditional birth attendants (TBAs), community health workers (CHWs), neighbourhood health committee (NHC) members, and women and husbands who are involved in maternal health programmes in the community. The SMAG members are specifically selected and trained to function as health promoters to deliver essential information on MHS to men and women in the community in order to create awareness and personal risk perception of pregnancy, labour and newborn health complications. Moreover, they encourage pregnant women to go for regular antenatal care (ANC) visits, delivery, and postnatal care (PNC) in a health facility. They are also trained to identify maternal and newborn complications during pregnancy, delivery, and the postnatal period, and to refer women with complications to health facilities for management [14–16]. Furthermore, the SMAG programme attempts to ensure involvement of husbands in MHS by encouraging men to participate as SMAG members [14–16]. Since SMAG members are selected by community members, specifically trained by healthcare staff to serve their local communities, and interact with both community members and healthcare facility staff, the SMAG programme aims to strengthen relationships between pregnant women and the healthcare facility staff. Thus, the SMAG programme aims to serve as a link between the community and the formal healthcare system on matters relating to maternal health [17–19].

Previous research conducted in Zambia and other developing countries including Ethiopia, Bangladesh and Pakistan has shown that community-centred interventions, such as SMAGs, that focus on community members’ involvement and participation are likely to be more accepted by local communities than vertical top − down interventions, which are planned by health workers at the national level and “imposed” on the community for adoption and implementation. Furthermore, community-based interventions are more likely to lead to desired health behavioural change and favourable health outcomes [14, 19, 20]. For example, Ensor and colleagues [14] reported that a community intervention in Zambia that focused on community participation led to an increased awareness of pregnancy-related complications and improved utilisation of MHS.

Nevertheless, few studies have explored the effectiveness of SMAGs in improving the utilisation of maternal and newborn health care services in low-income countries, including rural Zambia [16–18]. Currently, it is unknown how the community members in rural Zambia perceive the SMAG programme with regard to its effectiveness in increasing utilisation of facility-based MHS and improving maternal and newborn health outcomes. Therefore, this study aimed to explore the perceived SMAG programme’s effectiveness in increasing utilisation of maternal health services in Kalomo district, Zambia. This understanding is important as it will provide an evidence base for the design of national public health programmes and community interventions that focus on increasing use of facility-based delivery services and improving maternal and newborn health outcomes in the country.

## Methods

### Study design and setting

The study was qualitative in design and used in-depth interviews (IDIs) because the research team wanted to obtain individual information from various respondents and preclude any possibility of their answers (or opinions) being influenced by the presence of other informants as for example in a focus group discussio﻿n﻿ (FGD). The qualitative approach provides an in-depth understanding of the respondents’ experiences and beliefs concerning the SMAG programme which would in turn provide detailed understanding of the phenomenon under investigation [21, 22].

The study was conducted in Kalomo district, Zambia, a rural districts in the southern part of the country covering a total surface area of 15,000 km^2^. It has an estimated population of 275,779 [23–25] with an annual growth rate of 4.4%. Most of the population (92%) live in rural areas with subsistence farming and cattle rearing being the major economic activities. The district is one of the poorest in the country, with more than 70% of its population living on less than a dollar per day [25]. The health system comprises two hospitals, 40 health centres and an unrecorded number of health posts. Although most health centres provide basic emergency obstetric and newborn care (EmONC) services the district has low MHS utilisation rates, where less than 30% of the women receive assistance from a skilled birth attendant in a health facility, compared with 80% of births in urban women [2, 4, 5].

### Study population and sampling techniques

The study population consisted of SMAG members, nurses/midwives, district maternal and child health (MCH) coordinator, mothers, husbands, and local headmen. Local headmen were included in the study because the research team thought these would provide important information regarding the SMAG programme due to the influential role they play in the selection, coordination and monitoring of the SMAG members in the respective villages.

First, all district health centres which had trained and functional SMAGs were identified with the help of the district managers at the District Medical Office (DMO). Only ten out of thirty-six health centres in the district had functional SMAGs and all ten health centres were purposefully selected and included in the study. Next, selection of study participants within the health centre catchment area was done using a purposeful homogeneous sampling technique. This technique was used in order to select respondents who were familiar with the work of the local SMAGs, while, at the same time, allowing for recruitment of respondents with different characteristics and roles in the community in order to gain insight into the similarities and differences in their experiences [21, 22].

A month prior to the interview, the principal investigator contacted respective health centre in-charges to inform them about the study. The health centre in-charges were then asked to contact NHC members in their respective areas and to explain to them the purpose and objectives of the study. The NHC members were in turn asked to identify SMAG members, local headmen, mothers and fathers who were willing to participate in the study and to explain its purpose. The in-charges were also asked to identify nurses/midwives from their respective health centres who would be willing to participate in the study. NHC members were asked to inform the respondents who were willing to participate in the study to come to an agreed upon place − normally at the village headman’s residence − for the interview on an agreed upon date. The date for the interview was set by the health centre in-charge and then communicated to the research team through the principal investigator. The nurses/midwives were interviewed at their respective health centres.

#### The research instrument

A semi-structured interview guide was developed in English and translated into Tonga, the local language in the area. The interview guide comprised four pre-determined themes: 1) perspectives about the SMAGs (that is, their selection and training, respondents’ awareness about SMAGs, and respondents’ attitude towards SMAG members and the SMAG programme); 2) roles of SMAG members; 3) achievements of the SMAG programme; and 4) challenges facing the SMAG programme. Before developing the interview guide, the research team conducted a literature search to familiarise themselves with the SMAG programme. Following the literature review, the principal investigator developed a preliminary interview guide using a logic model which was based on the authors’ previous research findings [2, 4, 5] and the PRECEDE part in Green and Kreuter’s [26] PRECEDE/PROCEED model, which prescribes consideration of health-related behavioural determinants and environmental conditions at multiple levels [25]. The interview guide was shared with the other research team members. The document was also discussed with the district MCH coordinator during the district meeting. Following the district meeting, the document was revised and sent for translation into Tonga, by a bi-lingual expert. The initial document was in English because this is the official language in Zambia. Moreover, it was easy to discuss the document with the other research team members who were not familiar with the local language. The same interview guide was used for all the respondents. The semi-structured approach made it possible to make slight alterations to the questions depending on the answers provided by the participants and their specific roles in the community, while ensuring that the four central themes were covered.

### Data collection

The interviews were conducted between the 7th of April and 23rd of May 2015. To ensure privacy and confidentiality, each IDI was conducted in a quiet place, usually outside the local headman’s place, normally under a tree for shade. Each interview lasted between 30 and 50 min. The IDIs were conducted in Tonga and were facilitated by two trained research assistants; one conducted the interview, while the second one recorded the interview using a digital voice recorder. The principal investigator attended interviews at random to ensure that the research team members consistently followed the data collection protocol. Before each IDI, written consent was obtained from each participant by requesting them to read and sign the consent form, which had been translated from English into Tonga. Research assistants read the consent form aloud for those who could not read. Interviews with the nurses/midwives were conducted in English at the local health centre, normally in a quiet room or under a tree outside the health centre building.

After obtaining consent, research assistants requested each respondent to complete a short demographic questionnaire. The questions were read to the respondent in Tonga and answers recorded by the research assistants. The nurses/midwives read and filled in the demographic questionnaire themselves.

After interviewing 46 respondents (that is, 22 SMAG members, 5 nurses/midwives, 5 headmen, 10 mothers and 3 husbands) from the seven health centres, data saturation was achieved; that is, no more substantial information was obtained. At this point, the research team decided to stop the interviews and, thus, exclude the remaining three selected health centres.

### Data analysis

Demographic information was entered into an Excel sheet and transferred into IBM SPSS Statistics 21 for analysis. Descriptive statistics and frequencies were used to summarise the demographics of the respondents (Table 1). The voice recordings from the interviews were transcribed and translated into English by the research assistants. To check for accuracy, a few transcripts (20%) were back − translated into Tonga. Members of the research team then compared the Tonga and English versions for differences and similarities while listening to the original voice recording. After verification of translation accuracy, each transcript was then thoroughly read by one research assistant while the other one was listening to the corresponding voice recording. Each translated transcript was compared with the hand-written field notes that the research assistants had prepared during the interviews. After proof-reading and making corrections, the transcripts were saved on a password-protected computer. The word documents were then exported into Nvivo 10 MAC for processing. The exported data were then coded and categories and key sub-themes were identified. In order to make it easy to compare the perspectives of different respondents, the data from the five groups (that is, nurses/midwives, SMAG members, headmen, husbands, and wives) of respondents were coded separately. Data analysis was based on the four predetermined themes. An inductive approach was used to derive the sub-themes from the main themes by content-analysis and grouping all the similar statements made with respect to particular themes. Several sub-themes emerged from the data analysis; all sub-themes are described below in the respective sections for the main research themes.

**Table 1:** Demographic information

Variable	SMAGs (*n* = 22)	Nurses/midwives (*n* = 5)	Headmen (*n* = 5)	Husbands (*n* = 3)	Mothers (*n* = 10)
	Mean (SD)/*n*(%)	Mean (SD)/*n*(%)	Mean (SD)/*n*(%)	Mean (SD)/*n*(%)	Mean (SD)/*n*(%)
Age	46.56 (7.47)	33.5 (2.8)	54.4 (6.8)	28 (2.0)	25.6 (5.5)
Female	9 (40.9%)	100%	0 (0%)	0 (0%)	10 (100%)
Male	13 (59.1%)	0 (0%)	5 (100%)	0 (0%)	0 (0%)
Number of children	6 (2.8)	1.8 (2.1)	12.6 (6.8)	4.3 (4.1)	2.6 (2.1)
Marital status					
Married	16 (72.7%	2 (40%)	5 (100%)	3 (100%)	10 (100%)
Divorced	2 (9.1%)	0 (0%)	0 (0%)	0 (0%)	0 (0%)
Single	0 (0%)	2 (40%)	0 (0%)	0 (0%)	0 (0%)
Widow	4 (18.2%)	1 (20%)	0 (0%)	0 (0%)	0 (0%)
Level of education (in grades)					
Never attended	0 (0%)	0 (0%)	0	1 (33.33%)	0 (0%)
Lower primary (1-4)	0(0%)	0 (0%)	0	0 (0%)	0 (0%)
Upper primary (5-7)	6 (27.3%)	0 (0%)	3 (60%)	0 (0%)	5 (50%)
Junior secondary (8-9)	8 (36.4%)	0 (0%)	1 (20%)	1 (33.33%)	4 (40%)
Senior secondary (10-12)	8 (36.4%)	0 (0%)	1 (20%)	1 (33.33%)	1 (10%)
Tertiary	0 (0%)	5 (100%)	0 (0%)	0 (0%)	0 (0%)
Income level (in Zambian kwacha per month)					
Less than 100 K	12 (54.6%)	0 (0%)	3 (60%)	0 (0%)	5 (50%)
K100-K250	6 (27.3%)	0 (0%)	0 (0%)	1 (33.3%)	0 (0%)
K251-K500	6 (27.3%)	0 (0%)	2 (40%)	0 (0%)	3 (30%)
K501-K1000	0 (0%)	0 (0%)	0 (0%)	0 (0%)	0 (0%)
Above K1000	0 (0%)	5(100%)	0 (0%)	2 (66.7%)	2 (20%)
Occupation					
Housewife	2 (9.1%)	0 (0%)	0 (0%)	0 (0%)	3 (30%)
Unemployed	2 (9.1%)	0 (0%)	0 (0%)	0 (0%)	1 (10%)
Farmer	17 (77.3%)	0 (0%)	5 (100%)	3 (100%)	6 (60%)
Self-employed	1 (4.5%)	0 (0%)	0 (0%)	0 (0%)	0 (0%)
Formal employment	0 (0%)	5 (100%)	0 (0%)	0 (0%)	0 (0%)

Note: The maternal and child health (MCH) coordinator from the district medical office also participated in the study. She is not included in the analysis in this table

*SD* Standard deviation

## Results

Table 1 summarises the demographic characteristics of the 46 participants included in the study.

### Theme 1: Perspectives about SMAGs

#### Selection of the SMAG members

Respondents who were aware about the SMAG programme (that is all the respondents except for 2 headmen, 4 mothers and 1 husband) described SMAG members as community members who were chosen by the community in consultation with the headmen and healthcare staff from local health centres. They explained that SMAG members were chosen at a community meeting through voting. When selecting and voting for SMAG members, community members took into consideration whether the suggested people were respected, trusted and accepted by the community.

*“The SMAGs are chosen by the people. The nurses tell the headmen that we need people to become members of the SMAG group” (43 year old SMAG member).*

#### Training of SMAGs

Regarding training of the SMAG members, most respondents, including all SMAG members, nurses/midwives and 2 headmen confirmed that SMAG members were trained at the beginning of the programme in 2012 by the DMO with support from Zambia Integrated Systems Strengthening Project (ZISSP). They mentioned that the training took place only once, and that it focused on providing knowledge about safe motherhood and MHS.﻿

Additionally, 68% (15/22) of the SMAG members confirmed that the quality of the training was good and that it provided them with knowledge to identify danger signs during pregnancy and childbirth, and to help pregnant women in case of complications. However, the other 7 SMAG members and all 5 nurses/midwives complained that the training was too short and argued that they needed extra refresher training on how to manage labour complications.

*“The training was very short. There is need for training, refresher training” (38 year old female SMAG member).*

#### Awareness about SMAGs

All the respondents (except for 4 mothers, 2 headmen and 1 husband) were aware of the SMAG members and the work they did in their communities. They explained that they had either met SMAG members at community meetings in their villages or at their homes. Moreover, three of the six mothers who were aware of the SMAG programme mentioned that they had only met SMAG members at the health centre during ANC visits. One mother described SMAG members as:

*“SMAGs are members of that group which helps women the time they get pregnant until about 42 weeks after delivery to make sure that the mother and the baby are fine” (30 year old mother).*

#### Attitude towards SMAG members and the programme

Most respondents (excluding the 7 SMAG members and those respondents who were not aware of the programme) had a positive attitude towards the SMAG programme and the work the SMAG members do in the community.

In contrast, 3 headmen and all the nurse/midwives explained that in the beginning of the SMAG program, some community members including church leaders and husbands had a negative attitude towards the SMAG programme and that they did not accept the programme because they did not understand its purpose. In addition, some respondents (including the nurses/midwives, headmen and the two husbands who were aware of the SMAG programme) mentioned that some husbands were especially suspicious of the male SMAG members and did not allow their wives to participate in the community meetings. However, after the SMAG members explained the purpose of the SMAG programme and started teaching in the community, the community members started to understand and support the group.

*“The SMAGs are very good people, they are working well with the community and the government” (headman, 65 years old).*

### Theme 2: Roles of the SMAG members

The main roles of the SMAGs included organising community meetings to which they invited both pregnant and non pregnant women and their husbands. In these meetings they taught them about danger signs in pregnancy and childbirth and about the importance of attending ANC early in pregnancy. They also persuaded husbands to support their wives during pregnancy and to accompany them to the health centre for ANC visits. Moreover, SMAG members encouraged families to prepare for childbirth by saving money for the requirements for the mother and baby during labour and delivery at the clinic. Other roles included encouraging women to give birth at the clinic and to return for postnatal care. Further, respondents mentioned that the SMAG members move around the villages to monitor pregnant women and identify those who may have danger signs and need help. They encourage the women who live far from the clinic to go and wait for delivery at the mothers’ shelter at the clinic. In case of complications, respondents mentioned that SMAG members mobilise emergency transport and escort pregnant women to the health centre. Moreover, if the nurse was not available at the healthcare centre, SMAG members, specifically the former TBAs, would be called to go and assist pregnant women to deliver at the healthcare centre. Further, respondents mentioned that SMAG members hold meetings with stakeholders such as headmen, church leaders, and healthcare staff regarding the safe motherhood activities in their communities. They also write monthly reports about their community activities and send them to the healthcare centre and DMO.

### Theme 3: Achievements of the SMAG Programme

Most headmen, nurses/midwives, mothers and husbands who were aware of the SMAG programme believed that the SMAG programme had improved safe motherhood activities in the communities. For example, the nurse/midwives and headmen believed that the programme it led to an increase in community knowledge about danger signs and complications during pregnancy such as bleeding, high blood pressure, headache and fits, and to an increase in the importance of knowing and keeping in mind medical conditions during pregnancy such as nutrition, anaemia, and HIV. Furthermore, the nurses/midwives, headmen and mothers believed that the programme had led to an improvement in pregnant women’s attitude towards ANC use because they noticed that most women had started going early for ANC visits at the health centre. Moreover, respondents (especially the nurses/midwives, headmen, husbands and the SMAG members) explained that they had seen an increase in the number of husbands who accompanied their wives for ANC visits, HIV counselling and testing during ANC. They explained that those who were found to be HIV positive during ANC testing were put on treatment to prevent mother to child transmission of HIV during pregnancy. Respondents (especially the nurse/midwives and headmen) mentioned that they saw that many families started setting aside money for transport to the clinic and for buying requirements for the mother and baby during labour and childbirth (i.e. mother and baby clothes, soap and cleaning materials). Moreover, husbands, headmen and nurse/midwives explained that many pregnant women who lived far from the clinic started leaving home early to go and stay in the mothers’ shelter and wait for labour and childbirth from there. Moreover, all the nurse/midwives, SMAG members and the headmen mentioned that they had observed an increase in the number of women delivering at the health facility and those who returned for PNC at the health facility. All the nurses and headmen mentioned that the programme led to a reduction in the number of deaths for pregnant women and newborn babies.

In contrast, the five nurses/midwives complained that the increased number of pregnant women seeking health care at the health centres had resulted in a greater workload for the few available healthcare staff. In addition, the headmen confirmed that most health centres in their communities had few healthcare staff. The headmen, mothers and husbands complained that most health centres had no maternity waiting homes (MWHs) and that in those cases that they did exist, they were in a deplorable state. Furthermore, nurses/midwives complained that most health centres only had small labour wards which were not adequately equipped to handle emergency maternity cases.

Moreover, the mothers, husbands, headmen and SMAG members, mentioned that, many families found it difficult to find money to buy food to use when the woman stayed in the MWH. Others failed to save money to buy baby clothes that were needed during delivery at the health centre. Furthermore, some SMAG members and headmen mentioned that some socio-cultural norms and practices hindered many women from accessing health care from the clinics, and made them deliver at home and seek help from TBAs. Among these are trust in TBAs and the belief that women should not receive care from a male health worker. However, some respondents (especially the SMAG members and headmen) explained that the inclusion of TBAs in the SMAG programme helped to motivate most women to increase their confidence in the health centre’s services. They added that the SMAG programme motivated most women to put less trust in TBAs and home delivery. Rather, they motivated them to increase their trust in the nurses/midwives and to seek care from the health care facilities.

#### Theme 4: Challenges for SMAG members

Most (that is, 15 of 22) SMAG members mentioned that they were happy with their work because they helped save women’s lives. In contrast, the other 7 SMAG members complained that their voluntary work and lack of incentives demotivated most SMAG members as they had no time to do other work (for example, agricultural activities) and therefore they often had no income.

*“The work is good, although it is a voluntary work” (40 year old male SMAG member).*

Moreover, all the nurses/midwives and the 7 SMAG members complained that the lack of financial and material support from community members and the DMO made their work extremely difficult. In additionally, the headmen and all nurses/midwives mentioned that SMAG members did not have any means of finding transport to either reach pregnant women in the community or to take the women with complications to the health centre. They explained that some families (in particular, the single mothers) often failed to find money to pay for emergency transportation to the health centre. Furthermore, all the SMAG members and nurse/midwives confirmed that at the beginning of the programme﻿, SMAGs were given bicycles, which were shared among several SMAG members, but which also meant that some SMAG members did not receive anything and ended up walking long distances. Moreover, mothers and headmen explained that sometimes it was difficult for the community members to reach the SMAG members on phone because of the poor mobile phone network or dead batteries due to lack of electricity in many areas. One SMAG member explained that SMAG members had to pay for these calls from their personal resources.

*“The problem which they find is that they don’t have transport and where they work it is very far” (38 year old male SMAG member).*

All the nurses/midwives and headmen indicated that the number of SMAG members who were trained by the program was insufficient to reach the whole population in their respective catchment area.

Moreover, all the nurse/midwives and headmen mentioned that lack of refresher training demotivated most SMAG members. They confirmed that they only received training once at the beginning of the programme. Additionally, all the SMAG members confirmed that they needed training and learning materials (such as books, papers and pencils) in order to increase their knowledge.

## Discussion

This study aimed to explore the perceived SMAG programme’s effectiveness in increasing utilisation of MHS in Kalomo, Zambia. Overall, our findings show that most respondents were aware of the SMAG programme, knew who its members were, and had a positive attitude towards it. Moreover, most respondents believed that the SMAG programme led to an increase in awareness about pregnancy danger signs and an improvement in community attitudes towards MHS. Further, respondents believed that the SMAG programme led to an increase in ANC, facility-based delivery, and PNC utilisation rates. Moreover, the respondents felt that the programme resulted in an improvement in maternal and newborn health outcomes. However, respondents believed that various challenges affected implementation of the SMAG programme, such as lack of material and financial support to the programme, a lack of refresher training for SMAG members, and poor quality of care in health care facilities. Specifically, the respondents mentioned a lack of adequate MWHs, low health facility staffing levels, and the poor condition and small size of labour wards that were inadequately equipped to handle emergencies.

These findings are consistent with previous studies in Zambia [8, 9, 12, 14, 18, 20] which highlighted the importance of community-based interventions in improving women’s utilisation of MHS. They highlight the importance of engaging the community at every stage of the intervention development process to ensure programme success [26, 27]. These findings are consistent with the results of our previous study [12] and research from other countries [28], which showed that many pregnant women preferred using the services provided by community-based agents, such as TBAs, in whom they had trust and confidence because they were familiar with them and lived with them.

Second, these findings highlight the importance of the SMAG programme in increasing women’s awareness about danger signs in pregnancy and improving their attitude towards facility-based MHS such as ANC, delivery and PNC. For example, our findings suggest that women who had a positiveattitude towards facility-based health care ended up using these services. This finding is consistent with our previous studies [3, 12, 13] which showed the importance of women’s attitude towards facility-based health care services, as well as their past experience in influencing their intention to seek health care services. Moreover, the current study highlights the importance of socio-cultural norms − including the preferred place of delivery and the dependence on other family members such as friends, parents and husbands for decision-making and resources– in influencing health care seeking behaviour among pregnant women [3, 12, 13, 28] and highlights the need for public health interventions to consider the local cultural context when designing health promotion programmes.

Next, our findings highlight the importance of community involvement in the mobilisation of resources required to meet healthcare costs. For example, the current findings suggest that community sensitisation and door to door campaigns by the SMAG programme motivated many families to start preparing for childbirth in advance, by saving resources to meet the transportation costs and to buy the requirements for the mother and baby. Moreover, the current findings show that husbands started accompanying their wives to the health centre for ANC. Husbands also started allowing their wives to leave home to go and stay in the MWH and wait for labour. This finding is consistent with our previous studies [8–10, 12, 13] which reported the importance of family support, especially from the husband, in influencing pregnant women’s health seeking behaviour.

Further, our findings are consistent with our previous studies [8, 13], and align with WHO reports [6, 7, 10] that highlight the importance of facility-based skilled care in improving maternal and newborn health outcomes and showed that increasing supervised and facility-based deliveries in rural areas could improve maternal and newborn health outcomes in developing countries with a high MMR Moreover, this finding is importantbecause it provides a basis for public health interventions to promote access to and utilisation of facility-based health care services. However, since our findings are based on a qualitative study, there is a need to validate these findings using quantitative methods that will test whether communities with SMAGs perform better on use of MWHs than those without SMAGs.

However, our findings also highlight important contradictions between the SMAG programme goals and the current status of the health system. For example, our findings show that despite establishing an important SMAG programme that focuses on increasing demand for MHS provided in the healthcare facilities, the MoH did not address health system-related factors, such as constructing new MWHs and improving the quality of services in existing MWHs, expanding the size of labour wards and equipping them to be able to better handle complications, or increasing the number of nursing/midwifery staff. Moreover the SMAG programme faced various challenges, for example, respondents reported that training was only offered at the start of the programme, and the programme did not provide financial resources for emergency transport for women who developed complications or resources to buy the requirements for the baby [11]. This resulted in various management and logistical problems which affected the SMAG programme implementation, service provision and the quality of care that pregnant women received at the health care facilities. This finding is consistent with previous studies in Zambia [3, 4, 8, 9, 12, 13, 29] that report on the need for public health interventions to focus, not only on mitigating demand side factors, but also on supply side factors such as improving the quality of the MWHs and labour wards, increasing staffing levels and ensuring availability of medical equipment for emergency obstetric care in order to encourage women to use facility based healthcare [6–13, 29].

Some potential limitations to the present study should be noted. First, the findings focus solely on the experience of respondents recruited from the health centres where SMAG programmes were active, and respondents consisted of those who accepted to participate in the study. Therefore, the experiences of other community members from areas without SMAG members were not explored. Thus, the results might not be generalisable to individuals from communities/districts where SMAGs are not available. Moreover, some statements made by our respondents, for example on the decline in maternal and newborn mortality, cannot be verified without obtaining quantitative data from healthcare facilities and communities. Moreover, the first author’s experience as a former District Medical Officer for the research area might have influenced the results as respondents may have felt constrained in revealing their true opinions. Additionally, almost half (48%) of the respondents were SMAG members, which could have led to socially desirability bias in their responses as these respondents essentially evaluated their own activities. However, we believe that our use of an objective approach to the research process – by using independent trained research assistants, the rigorous data analysis process involving all members of the research team, and reporting responses from all respondents – makes our results representative and valid.

## Conclusion

The current findings show that, despite the various challenges affecting the SMAG programme and its implementation, respondents had a positive attitude towards the SMAG programme and believed that the programme led to an increase in the utilisation of MHS. These findings might provide a basis for the design of community-centered interventions focusing on increasing pregnant women’s demand for and utilisation of facility-based skilled MHS in rural Zambia. Furthermore, our findings suggest that the SMAG programme might be an important community intervention for improving maternal and newborn health outcomes in rural Zambia. However, our findings also suggest that before focusing on increasing demand for MHS provided in the health facilities, public health interventions need to focus on improving the quality of care provided in these healthcare facilities by: 1) constructing and improving the quality of MWHs and their services; 2) increasing the staffing levels for nurses/midwives; 3) constructing standard labour wards and adequately equipping them; and 4) improving the referral system.

## Abbreviations

ANC: Antenatal care
CHWs: Community health workers
DMO: District Medical Office
IDIs: In-depth interviews
MCH: Maternal and child health
MMR: Maternal mortality ratio
MOH: Ministry of Health
MWHs: Maternity waiting homes
NHCs: Neighbourhood Health Committees
PNC: Postnatal care
SMAGs: Safe Motherhood Action Groups
SPSS: Statistical Package for Social Sciences
TBAs: Traditional birth attendants
